# Adsorption and Dissociation of Ni(acac)_2_ on Iron by Ab Initio Calculations

**DOI:** 10.1021/acs.jpca.0c05040

**Published:** 2020-09-03

**Authors:** Chiara Corsini, Stefan Peeters, M. C. Righi

**Affiliations:** † Department of Physics and Astronomy, Alma Mater Studiorum University of Bologna, Via Berti Pichat 6/2, 40127 Bologna, Italy; ‡ Department of Physics, Informatics and Mathematics, University of Modena and Reggio Emilia, I-41125 Modena, Italy

## Abstract

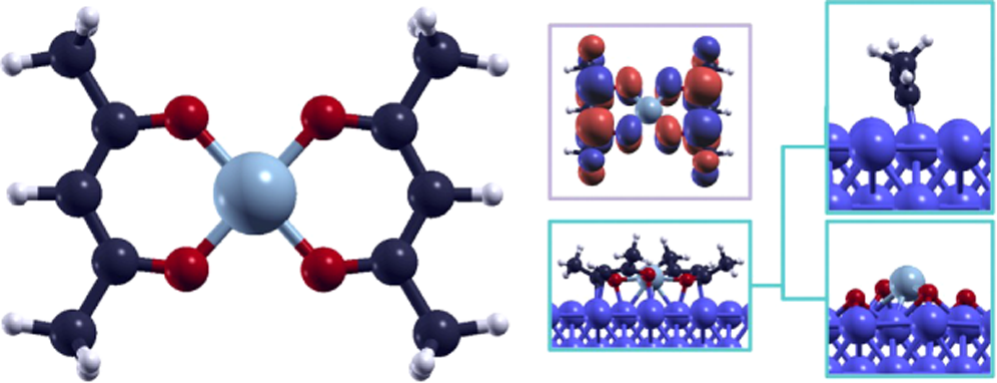

Among metal β-diketonates, nickel acetylacetonate (Ni(acac)_2_) has been widely employed as a precursor for many chemical structures, due to its catalytic properties. Here, we investigate, by means of density functional theory (DFT) calculations, the adsorption and dissociation of this complex: after an evaluation of the structural and electronic properties of Ni(acac)_2_, a comparison between different dissociation patterns reveals that the most favorable pattern for the complex adsorbed on iron is different from the one suggested by considering the strength of the bonds in the isolated complex and an attempt to generalize this dissociation model is made in this work. Moreover, the most favorable adsorption configurations turned out to be a *long bridge* positioning of the nickel atom along with an *on top* positioning of the oxygen atoms of Ni(acac)_2_, while a *short bridge* positioning is the most favorable for the central metallic unit alone.

## Introduction

Metal β-diketonates are a class of metallorganic compounds widely employed as chemical precursors for their volatility, rich reactivity, and commercial availability. Because of their catalytic properties, these compounds are ideal for several applications.^1^ In fact, nickel acetylacetonate, Ni(acac)_2_, a member of this group of complexes, is employed in optoelectronics,^2,3^ electrocatalysis,^4^ polymer chemistry,^5,6^ film growth,^7,8^ synthesis of nanoparticles,^9^ and nanocomposites.^10−12^

However, the properties of Ni(acac)_2_ adsorbed on iron are still scarcely described in the literature. In 1967, Kishi and co-workers proposed a description of the adsorption of acetylacetonate units on nickel and iron, based on infrared and ultraviolet spectroscopies.^13^ The adsorption and the decomposition of Ni(acac)_2_ on a metallic substrate were never investigated, to the best of our knowledge. Understanding the atomistic mechanism of the dissociation of this complex on metal can be useful to clarify its functionality. Here, we present a study based on density functional theory (DFT) concerning the adsorption and dissociation of Ni(acac)_2_ on iron. After presenting structural and electronic properties that were never calculated before for isolated Ni(acac)_2_ by means of DFT, we present in this work the adsorption and dissociation energies of this complex on different sites of the iron surface.

## Methods

**Figure 1:**
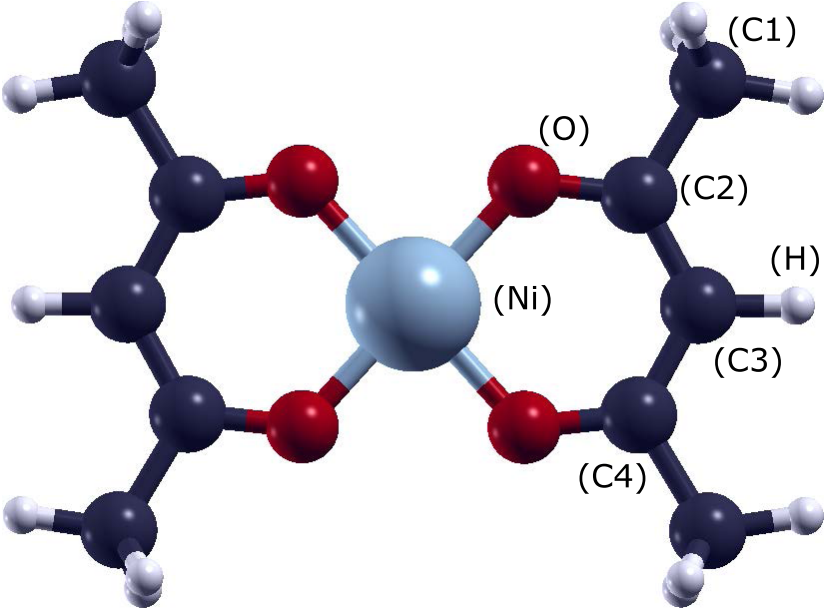
Chemical structure of Ni(acac)_2_.

We focused on the monomeric form of the Ni(acac)_2_ complex shown in Figure 1.

To study the properties of Ni(acac)_2_ and its interaction with an iron surface, the Quantum Espresso package^14,15^ was used to perform first-principle calculations within the framework of DFT. The Perdew, Burke, and Ernzerhof (PBE) approximation^16^ was used to describe electronic exchange and correlation. The plane wave/pseudopotential approach was employed, with cutoffs of 35 and 280 Ry applied to the kinetic energy of the wave functions and the charge density, respectively, as the pseudopotentials were ultrasoft. The choice for these values of the cutoffs is justified in the Supporting Information. Spin polarization was used and smearing with Gaussian functions was applied with a width of 0.02 Ry to better describe electronic occupations around the Fermi level. For the geometry optimization of the isolated complex, a cubic supercell of 60 Å was used to avoid interactions between the periodic replicas. The adsorption of the molecule on iron was studied considering the (110) surface, as it is the most stable surface for this metal.^17,18^ A supercell of iron with a 5 × 3√2 in-plane size and a height 6√2 times the lattice parameter of bulk iron was used, containing a slab composed of four layers with 30 atoms each. Integrations were carried out at the Γ point for the calculations in the cubic cell, while for the orthorhombic cell, a 2 × 2 × 1 Γ-centered grid was chosen. An initial guess for the geometry of the complex was obtained with the software Avogadro.^19^ The molecular structure was then optimized, with the total energy and the forces converged under the thresholds of 10^–4^ and 10^–3^ Ry, respectively.

## Results and Discussion

### Properties of Isolated Ni(acac)_2_

The chemical structure of Ni(acac)_2_ obtained from the geometry optimization agrees with the other existing data in the literature.^20−22^ Calculated bond lengths and angles, reported in Table 1, differ no more than 2 and 3% in absolute value, respectively, compared to previous experimental and theoretical observations. The symmetry of the complex was also verified by analyzing the discrepancies among equivalent bonds. Such discrepancies were always below 0.1%, which is a low enough value to reasonably confirm the symmetry of the chemical structure.

**Table 1:** Bond Lengths and Angles of Ni(acac)_2_^a^

bond	length (Å)	angle	size (deg)
Ni–O	1.85	O–Ni–O	95.9
O–C2	1.28	Ni–O–C2	125.6
C2–C3	1.40	O–C2–C3	125.0
C2–C1	1.51	O–C2–C1	114.5
C3–H	1.09	C2–C3–C4	122.9

a The atomic labels correspond to the ones shown in Figure 1.

Electronic properties of the Ni(acac)_2_ complex were also evaluated. The ionization energy *E*_*i*_ was calculated as
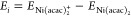
1where *E*_Ni(acac)_2_^+^_ and *E*_Ni(acac)_2__ are the total energies of the cationic complex obtained by removing one electron and of the neutral complex, respectively. The calculated value of 6.25 eV is comparable to the experimental one of 7.41 eV,^23^ with an underestimation of about 16%, most probably due to the well-known problem of the fundamental gap in DFT.^24^

**Figure 2:**
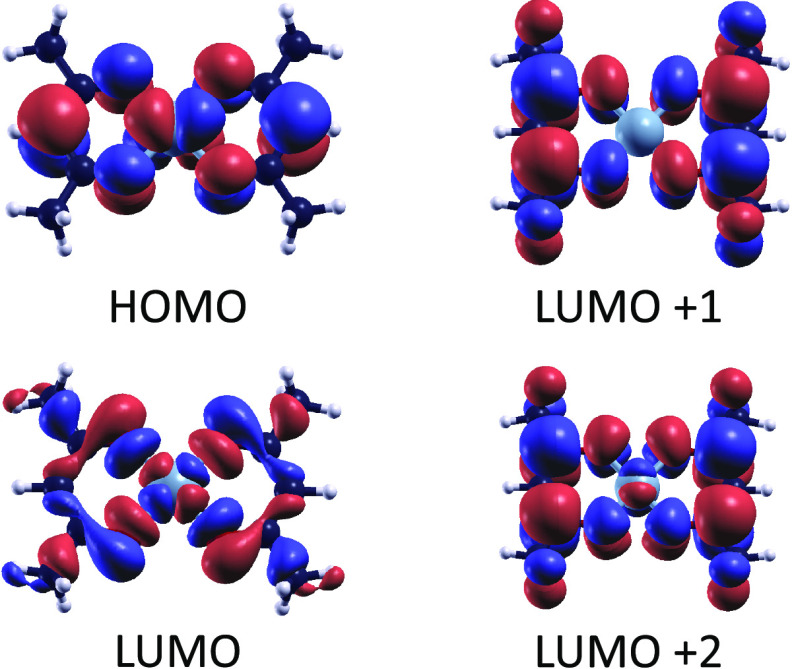
Frontier molecular orbitals of Ni(acac)_2_ plotted with an isovalue of 0.003. The red and blue colors of the isosurfaces identify the positive and negative signs of the wave function, respectively.

The frontier molecular orbitals of Ni(acac)_2_ are shown in Figure 2. The lowest energy state corresponds to a singlet state in which the highest occupied molecular orbital (HOMO) is the result of a combination of the *d*_*xz*_ orbital of Ni, accounting for almost 70% of the wave function, and one *p*_*z*_ orbital for each of the O and C3 atoms, collectively accounting for more than 20% of the wave function. The lowest unoccupied molecular orbital (LUMO) is composed of almost 60% of the Ni *d*_*xy*_ orbital and of the O *p*_*y*_ orbitals, which collectively account for almost 25% of the wave function. LUMO + 1 and LUMO + 2, which are mostly obtained by combining *p*_*z*_ orbitals of the C and O atoms of the ligand units, are in perfect agreement with the plots proposed in the literature for the LUMO and LUMO + 1.^25^ In fact, the high number of nodal planes in the lowest unoccupied molecular orbital (LUMO) may suggest that the calculated energy of this orbital is underestimated, and it should lie above LUMO + 2.

**Figure 3:**
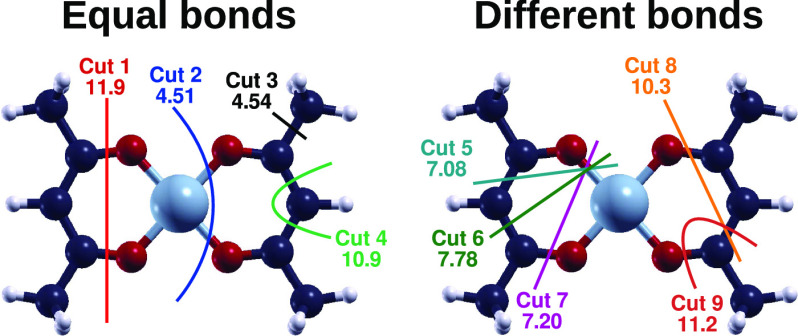
Fragmentation patterns considered for Ni(acac)_2_, divided into two groups depending on whether equal or different bonds are broken. An exception is Cut 3, in which a single bond is broken, compared to all of the other patterns in which two bonds are broken. The fragmentation energies, calculated as in eq 2 and expressed in eV, accompany each pattern.

To estimate which could be the most favorable dissociation pattern for the isolated complex, the fragmentation energy *E*_frag_ was determined as
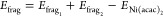
2where *E*_frag_1__ and *E*_frag_2__ are the total energies of the individual fragments obtained after molecular dissociation. This analysis aims at comparing different ways of breaking the molecules to evaluate the strength of the broken bonds. The absolute values of fragmentation energies obtained in eq 2 cannot represent realistic dissociative events, where the fragments can rearrange and can be stabilized by the presence of other chemical species. However, it is instructive to compare the fragmentation energies of different possible fragmentation patterns to understand which are the strongest bonds in the complex. We considered nine different fragmentation patterns in total, shown in Figure 3. These patterns do not exhaust all of the possible combinations for the molecular dissociation; yet, they were chosen among the most representative cuts for this complex.

The lowest fragmentation energy is observed in the case of Cut 2, where two Ni–O bonds are broken. Breaking two C–C bonds is more difficult, requiring 5.8 (Cut 8) to 7.4 (Cut 1) eV more than Cut 2. As expected, the fragmentation energies of mixed patterns where a Ni–O bond and a C–C bond are broken lie in the middle. Therefore, Cut 2 may be the most likely to occur for isolated molecules, as the energy required to separate the two fragments is lower with this scheme. However, the effect of a metallic substrate can change the picture provided by these calculations, as the fragments of the molecules can be stabilized differently on the surface of the metal, in favor of a dissociative pattern that involves the stronger C–O bonds.

One could roughly estimate the strength of individual bonds in the complex by considering half of the fragmentation energies of Cuts 1, 2, and 4. This calculation would yield 5.96, 2.25, and 5.46 eV for C–O, Ni–O, and C–CH bonds, respectively. Cuts 5–9 are indeed the combination of these fragmentation patterns and summing up the fragmentation energies of the individual bonds would yield 7.71, 8.21, and 11.4 eV for Ni–O + C–CH (Cuts 5 and 6), Ni–O + C–O (Cut 7), and C–O + C–CH (Cuts 8 and 9), respectively. These last values differ from the energies on the right side of Figure 3. Such discrepancies are most probably due to the different electronic structures of the fragments, which are left to relax to the minimum energy configurations in all of the calculations. Constraining the electronic configurations by assigning specific spin multiplicities to the fragments would probably provide better agreement between the calculated bond strengths; yet, such a detailed analysis goes beyond the purpose of this study, as a qualitative consistency can already be observed in the fragmentation energies calculated with the present approach.

### Adsorption and Fragmentation on Iron

**Figure 4:**
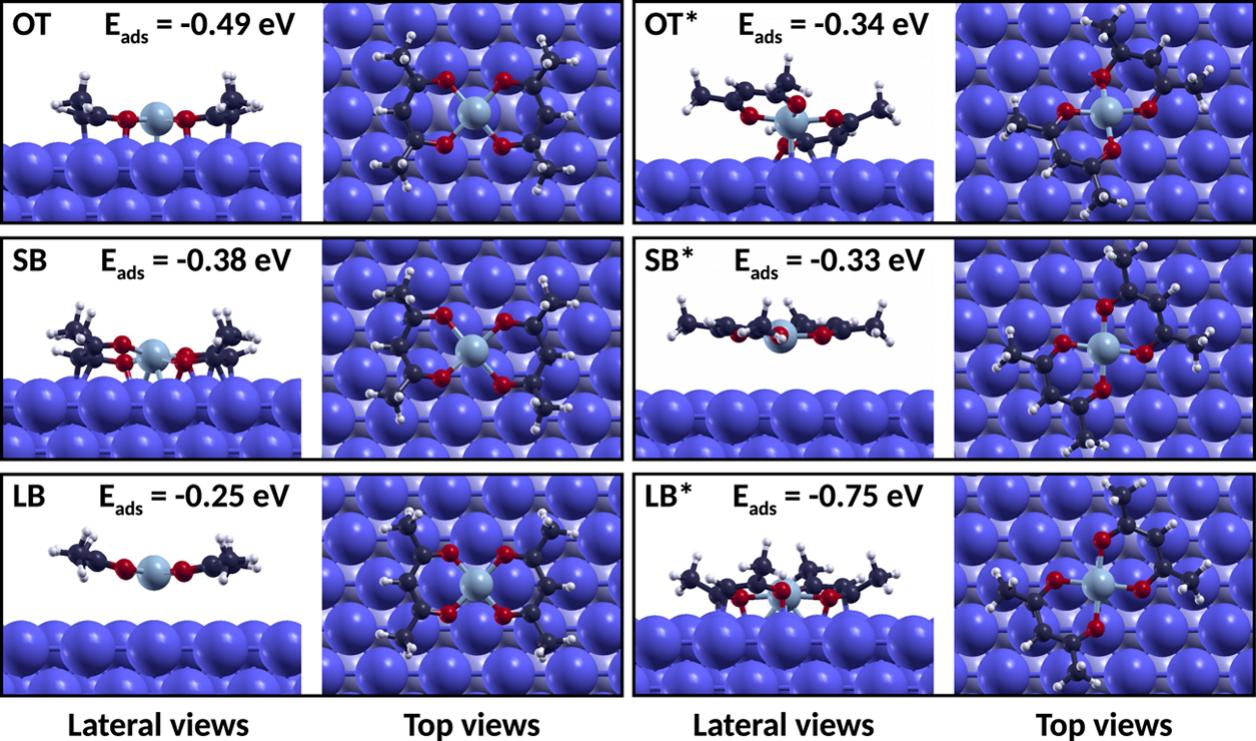
Optimized geometries of Ni(acac)_2_ on the iron (110) surface, with the complex placed in the *on top* (OT), *short bridge* (SB), and *long bridge* (LB) configurations and their rotated counterparts, marked by an asterisk, along with the respective adsorption energies. The size of the blue-colored iron atoms was increased by 50% in the pictures. In the top views, the iron atoms below the first layer were colored with a lighter blue to better distinguish the relevant sites on the surface.

To better understand the effect of a metallic substrate on the chemical properties of Ni(acac)_2_, the Ni(acac)_2_ complex was adsorbed on an iron (110) surface. Three nonequivalent sites of the surface were chosen as the initial position of the Ni atom of the complex: the position *on top* of an iron atom (OT), the twofold coordination site, called the *short bridge* (SB), and the fourfold coordination site, called the *long bridge* (LB). Two different orientations of the complex were considered for all three sites: with the ligand units parallel to the *y*-axis of the supercell (OT, SB, and LB) and with the complex rotated by 45° (OT*, SB*, and LB*). During the geometry optimization of the complexes on the iron surfaces, the *x* and *y* coordinates of the Ni atom were kept fixed, while all of the other degrees of freedom were allowed to relax. The final configurations of the adsorbed systems are shown in Figure 4.

When the complex was in the OT, OT*, SB, and LB* configurations, the complex chemisorbed on the iron surface, while in the case of the SB* and LB configurations, only physisorption could be established. For all of these systems, the adsorption energy *E*_ads_ was evaluated as
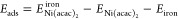
3where *E*_Ni(acac)_2__^iron^ and *E*_iron_ are the total energies of the system composed by the complex adsorbed on the iron slab and of the iron slab alone, respectively. The adsorption energy for each configuration is included in Figure 4. One of the most favorable configurations for the Ni atom on the surface is the OT position. However, the LB* configuration is especially favorable because all of the O and C3 atoms are almost on top of iron atoms, increasing the number of bonding interactions and leading to a more stable configuration. In fact, rotating the complex by 45° is favorable only when the Ni atom is on the LB site. In the other cases, instead, the rotated complex cannot efficiently adsorb on the surface, resulting in twisted geometries (OT*) or weak physisorption (SB*).

### Bond Dissociation on Iron

**Figure 5:**
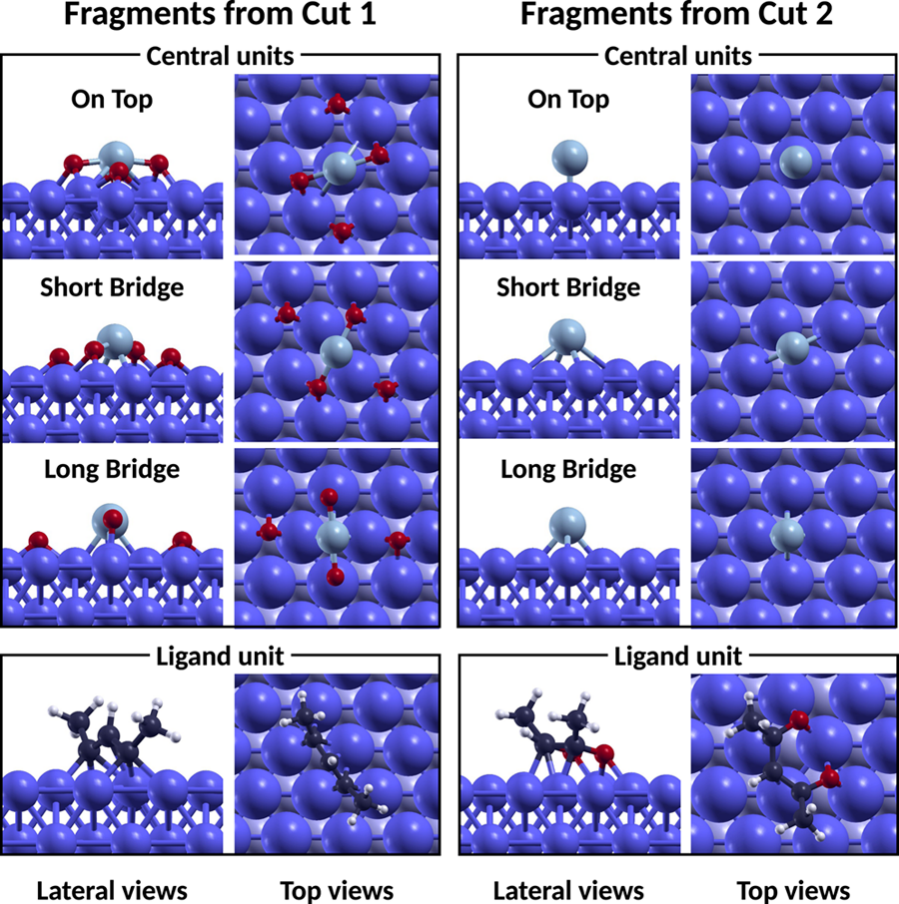
Optimized geometries of the fragments of Ni(acac)_2_ obtained from Cut 1 and Cut 2 on the iron surface. In the top views, the sizes of all of the iron atoms were increased by 50% and the atoms below the first layer were colored with a lighter blue.

The metallic substrate can play an important role in modifying the mechanisms of molecular dissociation. To verify whether the dissociation mechanism predicted for isolated Ni(acac)_2_ is still the most favorable also on iron, we calculated the dissociation energies on the substrate *E*_frag_^iron^ as
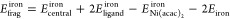
4where *E*_central_^iron^ and *E*_ligand_^iron^ are the total energies of the fragments corresponding to the central metallic unit and to a single ligand unit, respectively, adsorbed on the iron surface. The total energy of the iron slab must be subtracted twice in eq 4 to balance the number of iron atoms. The dissociation energies on iron were calculated for different placements of the metallic centers and ligand units originated by Cut 1 and Cut 2. Lateral and top views of the fragments considered are included in Figure 5. The sites and orientations considered for the central metallic units were the most favorable found in the adsorption calculations, namely, OT, SB, and LB*. Three different orientations were considered for each of the ligand units, which were left to relax freely on the surface. Each ligand unit was placed on the iron surface with the carbon chain parallel to the *x-*axis, rotated by 45°, and parallel to the *y*-axis, to explore different adsorption configurations on the surface. For both the fragments, the most favorable configurations turned out to be the ones in which the units were rotated by 45°. These adsorption configurations, shown in the bottom panels of Figure 5, were the ones considered for the calculation of the dissociation energy in eq 4.

Table 2 reports the calculated dissociation energies of the Ni(acac)_2_ complex on the iron surface. All of the calculated dissociation energies are negative, meaning that the dissociation of the complex is favorable on the substrate. Cut 1 is the most favorable way of breaking the complex, as the energies obtained from this pattern are higher in absolute value than the ones of Cut 2. This result shows that the preferred dissociation pattern changes from Cut 2 for the isolated molecule to Cut 1 for the complex adsorbed on the iron substrate. The fragments obtained upon dissociation on the iron surface can be stabilized differently with respect to the isolated molecular fragments, which are unable to relax their strained geometry. In particular, for Cut 1, such favorable values of dissociation energies are also due to the detachment of the oxygen atoms of the central metallic cores, leading to significantly higher stability of the system. For both dissociation patterns, the most favorable destination for the metallic core is the SB position, providing the lowest (highest in absolute value) dissociation energy.

**Table 2:** Reaction Energies for the Dissociation on the Iron Substrate *E*_frag_^iron^ in eV

	OT	SB	LB*
Cut 1	–1.88	–2.70	–1.62
Cut 2	–0.82	–1.49	–1.27

Since only part of the O atoms detached from the Ni atom in the central units originated by Cut 1, we verified whether the full dissociation of the NiO_4_ unit on iron is thermodynamically favorable, by calculating the following reaction energy
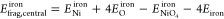
5where *E*_frag,central_^iron^ is the energy required to fully divide the NiO_4_ central unit and *E*_Ni_^iron^, *E*_O_^iron^, and *E*_NiO_4__^iron^ are the total energies of the respective isolated species, all adsorbed on iron. The values of *E*_frag,central_^iron^ vary by changing the adsorption site of the Ni atom on the surface; yet, they range from −1.33 (SB) to −2.20 eV (LB), with the negative sign indicating that the full dissociation of the NiO_4_ central unit is favorable and that the final product of the dissociation will most probably consist of Ni and O atoms fully separated on the iron surface, as long as enough energy is provided to the system to overcome the dissociation barriers.

In a previous study regarding molybdenum dithiocarbamates, we discovered that the most probable fragmentation pattern for these complexes on iron differs from the one for the isolated complexes, predicted by analyzing the strength of the bonds that take part in the dissociation.^26,27^ The results of the fragmentation analysis are in perfect agreement with the ones obtained in this work for Ni(acac)_2_ and they suggest the following general rules for small metallorganic complexes interacting with the iron surface:

1. While the molecular dissociation of the isolated complexes is energetically unfavorable, the iron substrate is capable of stabilizing the fragments of the dissociation, turning it into a favorable process.
2. The dissociation pattern in which the weakest bonds of the isolated complexes are broken is not the most probable when the complexes are on the iron surface. Alternative paths can be observed in which stronger bonds are broken, due to the stabilization of the relative fragments by the substrate.

In fact, Ni(acac)_2_ and most of the MoDTC structures on iron can be broken more preferably between the chalcogen atoms and the adjacent carbon atoms of the ligand units. These chalcogen atoms remain near the central metallic units, completing the coordination of the metal, and possibly detach in a subsequent step, while the carbon atoms with broken bonds strongly bind to the iron surface.

## Conclusions

We presented a study concerning the interaction of Ni(acac)_2_ with an iron surface by means of DFT. After confirming the good agreement between the optimized geometry of the complex with the existing structural data available, we calculated the frontier orbitals and the ionization energy of this complex for the first time with DFT. A fragmentation analysis both for the isolated complex and for the complex adsorbed on iron was performed and different adsorption sites were considered to understand which is the most preferable configuration of Ni(acac)_2_ on the iron surface. The main results are summarized as follows:

- For isolated Ni(acac)_2_, the most favorable fragmentation pattern is *Cut 2*, corresponding to the dissociation of the acetylacetonate ligand units from the nickel atom.
- The most favorable adsorption configuration for Ni(acac)_2_ on the iron surface is LB*, requiring the nickel atom to be in the *long bridge* position and the oxygen atoms to be on top of the nearest iron atoms. After dissociation, the central metallic unit of the complex is more stabilized in the *short bridge* position.
- Both dissociation patterns, which are not favorable for the isolated complex, become favorable on iron. The most favorable fragmentation pattern for the complex adsorbed on iron becomes Cut 1 due to the stabilization effect of the substrate. These observations, in agreement with the ones drawn for MoDTC complexes, suggest a possible generalization of the dissociation mechanism on the iron surface for small metallorganic complexes where the ligand units are connected to the metallic center through chalcogen atoms.

The reaction energies for the fragmentation calculated in this work do not take into account the energy barriers associated with the dissociation. A further step of this study could include the estimation of such barriers and the dynamic simulations of the dissociation, to better understand the kinetics of the different mechanisms.

## Supporting Information Available

Details of the cutoff for the kinetic energy of the wave functions (PDF)
