# Supplementary material for: Adsorption and Dissociation of Ni(acac)2 on Iron by Ab Initio Calculations
Source: J Phys Chem A. 2020 Sep 3;124(39):8005–10. doi: 10.1021/acs.jpca.0c05040 (PMC8011918; doi:10.1021/acs.jpca.0c05040)
Supplement: Supplementary file 1 — jp0c05040_si_001.pdf [file jp0c05040_si_001.pdf]

Supporting Information: Adsorption and Dissociation of $Ni(acac)_2$ on Iron by Ab Initio Calculations

Chiara Corsini,† Stefan Peeters,†,‡ and M. C. Righi*,†

†*Department of Physics and Astronomy, Alma Mater Studiorum University of Bologna, Via Berti Pichat 6/2, Bologna, Italy*

‡*Department of Physics, Informatics and Mathematics, University of Modena and Reggio Emilia, I-41125 Modena, Italy*

E-mail: mariaclelia.righi2@unibo.it

Convergence test

In order to choose a proper value for the cutoff on the kinetic energy of the wave functions, a convergence test was carried out by taking into account the ionization energy of the molecule, which is a property directly comparable with the data available in literature. The ionization energy was calculated at different energy cutoffs according to Equation 1 in the main text. The ionization energy was found at convergence below 0.3 meV/atom for a cutoff of 35 Ry, as shown in Table S1 in the present document. A plot of the convergence test is shown in Figure S1 in the present document.

Table S1: Kinetic energy cutoffs (E_{cut}) in Ry, ionization energies (E_i) in eV and the differences in ionization energies between two adjacent lines (ΔE_i) in $eV/atom$.

E_{cut}	E_i	ΔE_i
25	6.380726083	0.00451
**35**	**6.250050137**	**0.00022**
45	6.243631648	0.00001
55	6.243631648	0.00010
65	6.240372947	

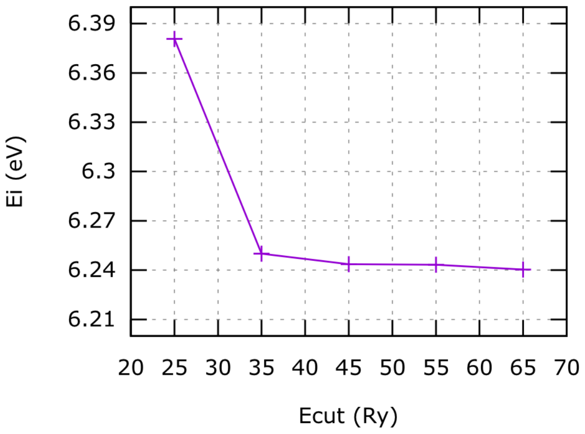

Figure S1: Ionization energy values (eV) calculated at different kinetic energy cutoffs (Ry).
